# Supplementary material for: Predictors of health facility childbirth among unmarried and married youth in Uganda
Source: PLoS One. 2022 Apr 7;17(4):e0266657. doi: 10.1371/journal.pone.0266657 (PMC8989320; doi:10.1371/journal.pone.0266657)
Supplement: Attachment — Submitted filename: Reviewer feedback_MM.docx [file pone.0266657.s001.docx]

REVIEW PLOS ONE

TITLE: FACTORS ASSOCIATED WITH HEALTH FACILITY DELIVERY AMONG UNMARRIED AND MARRIED YOUTH IN UGANDA

ARTICLE ID: PONE-D-20-31194

**General comments**

1. The finding of this study is pertinent and informative to policy makers since it was based on national DHS data.
2. Well written and informative. But the authors have used many studies as reference (76) meaning there are too many studies on the topic or the authors are unable to use quality studies as reference by critically appraising for quality. Please comment on these issues

**Abstract**

3. The abstract is not organized as per the PLOS ONE journal guideline. Please try to organize the abstract as per the journal guideline.
4. The relevance of including line 8 “Most factors were consistent with patterns observed in the literature among youth” is not clear. It is not the part of your finding rather you compared your findings with previous literature. Better to remove it.

**Background**

5. The background is well presented
6. But it is too much which needs to be summarized without compromising its content.

**Methods: Well but there are some issue on analysis method to be addressed**

7. While assessing the independent variables, there are “individual level” and “community level” factors. It would be better if the authors consider “multilevel logistic regression analysis” instead of “logistic regression analysis” since there might be variation across the level of factors
8. The numbers of variables entered in the multivariable model are many. How was the stability of the multivariable model?
9. Univariate, bivariate, and multivariate analysis were performed. Please clearly report the criteria to include variables in the multivariable model in “the statistical analysis” of the method section. Have you include all variables entered to the bivariable model for the multivariable model too?
10. The authors used both “Pearson chi-square” line 177 and “binary logistic regression” lines 179-180 to report bivariate analysis. Why the authors prefer to report both Pearson chi-square and binary logistic regression at the same time for bivariate analysis? What is the relevance of using “Pearson chi-square” in combination to “binary logistic regression” for bivariable analysis?

**Results:**

Overall the result is well done. But the description is too large which needs to be summarized.

11. The descriptive characteristics of the respondents from page 10, lines 207-216 is a bit too much. Better to summarize it since already presented in the table.
12. In statistical report there is an agreement that the description of table shall be presented above the table but in the case of this paper (e.g. table 2), the description of the table is also reported below the table which is not recommended. Please try to correct this issue.
13. Table 3 needs to be organized in easily understandable manner for readers since the variables, reference category and statistical values are not reported clearly.

**Discussion**

The discussion is good.

**Conclusion**

14. The conclusion in the abstract and the main document are not consistent. Please try to address this issues.
15. Good if the authors clearly report factors that positively and negatively associated with facility delivery utilization in the conclusion section of abstract as well as the main document.
