# Supplementary material for: Predictors of health facility childbirth among unmarried and married youth in Uganda
Source: PLoS One. 2022 Apr 7;17(4):e0266657. doi: 10.1371/journal.pone.0266657 (PMC8989320; doi:10.1371/journal.pone.0266657)
Supplement: Attachment — Submitted filename: Response to Reviewers.docx [file pone.0266657.s002.docx]

REVIEW PLOS ONE

TITLE: FACTORS ASSOCIATED WITH HEALTH FACILITY DELIVERY AMONG UNMARRIED AND MARRIED YOUTH IN UGANDA

ARTICLE ID: PONE-D-20-31194

| **No.** | **Reviewers’ comments** | **Authors Response** |
| --- | --- | --- |
|  | **Reviewer** |  |
| 1 | The finding of this study is pertinent and informative to policy makers since it was based on national DHS data. | Thank you for the compliment |
| 2 | Well written and informative. But the authors have used many studies as reference (76) meaning there are too many studies on the topic or the authors are unable to use quality studies as reference by critically appraising for quality. Please comment on these issues | We have appraised the studies and used quality studies to justify the need for our study |
| 3 | The abstract is not organized as per the PLOS ONE journal guideline. Please try to organize the abstract as per the journal guideline. | We have organized the abstract to clearly show the different sections as per PLOS ONE journal guidelines |
| 4 | The relevance of including line 8 “Most factors were consistent with patterns observed in the literature among youth” is not clear. It is not the part of your finding rather you compared your findings with previous literature. Better to remove it. | This sentence has been removed |
|  | **Background** |  |
| 5 | The background is well presented | Thank you for the compliment |
| 6 | But it is too much which needs to be summarized without compromising its content. | We have tried to summarise the background of the study |
|  | **Methods** |  |
| 7 | While assessing the independent variables, there are “individual level” and “community level” factors. It would be better if the authors consider “multilevel logistic regression analysis” instead of “logistic regression analysis” since there might be variation across the level of factors | We explored the use of multilevel modeling but the small sample of youth in each cluster could not allow the use of cluster as the second level of analysis.  This is because the Uganda Demographic and Health Surveys sampling is random by cluster and household levels. These would be the perfect levels for the multilevel analysis but both have few cases. |
| 8 | The numbers of variables entered in the multivariable model are many. How was the stability of the multivariable model? | We paid careful attention to stability of the models and assessment of potential multicollinearity, given potential correlation of some of the predictor variables (e.g. education, wealth index, occupation, etc). An examination of changes in parameter estimates and standard errors as predictor variables were added to the model in successive stages showed no evidence of model instability. |
| 9 | Univariate, bivariate, and multivariate analysis were performed. Please clearly report the criteria to include variables in the multivariable model in “the statistical analysis” of the method section. Have you included all variables entered to the bivariable model for the multivariable model too? | We have clarified this and its variables that were significant in either group at bivariate level that were included in the multivariate models |
| 10 | The authors used both “Pearson chi-square” line 177 and “binary logistic regression” lines 179-180 to report bivariate analysis. Why the authors prefer to report both Pearson chi-square and binary logistic regression at the same time for bivariate analysis? What is the relevance of using “Pearson chi-square” in combination to “binary logistic regression” for bivariable analysis? | This was a mistake and we have corrected it; Pearson chi-square was used for bivariate analysis and logistic regression was used for multivariate analysis to find the predictors of health facility delivery among the youth |
|  | **Results** |  |
| 11 | The descriptive characteristics of the respondents from page 10, lines 207-216 is a bit too much. Better to summarize it since already presented in the table. | Thank you, this has been summarised |
| 12 | In statistical report there is an agreement that the description of table shall be presented above the table but in the case of this paper (e.g. table 2), the description of the table is also reported below the table which is not recommended. Please try to correct this issue. | This has been corrected throughout the paper |
| 13 | Table 3 needs to be organized in easily understandable manner for readers since the variables, reference category and statistical values are not reported clearly. | Table three has been organized in a clearer and easily understandable way as we have separated the variables and reference categories. Odds ratios and corresponding 95% Cis are also put in one cell |
|  | **Conclusion** |  |
| 14 | The conclusion in the abstract and the main document are not consistent. Please try to address this issue. | This has been addressed and the conclusion in the abstract and main document are consistent |
| 15 | Good if the authors clearly report factors that positively and negatively associated with facility delivery utilization in the conclusion section of abstract as well as the main document. | Factors that are positively and negatively associated with the outcome variable are pointed out in the conclusion sections |
|  | 2. We note you have included a table to which you do not refer in the  text of your manuscript. Please ensure that you refer to Table 1 in your text; if accepted, production will need this reference to link the reader to the Table. | I have referred to table 1 in the text in line 141 in the clean copy of the manuscript |
| Reviewers' comments: |  |  |
|  | 1. Is the manuscript technically sound, and do the data support the  conclusions?  The manuscript must describe a technically sound piece of scientific research with data that supports the conclusions. Experiments must have been conducted rigorously, with appropriate controls, replication, and  sample sizes. The conclusions must be drawn appropriately based on the  data presented.  Reviewer #1: Yes  Reviewer #2: Partly | We believe that the revisions in the manuscript have addressed concerns raised by Reviewer 2 |
|  | 2. Has the statistical analysis been performed appropriately and  rigorously?  Reviewer #1: Yes  Reviewer #2: No | Reasons for the use of binary logistic regressions and not multilevel models has been explained in number 7 above |
|  | 3. Have the authors made all data underlying the findings in their manuscript fully available?  The PLOS Data policy [2] requires authors to make all data underlying the findings described in their manuscript fully available without restriction, with rare exception (please refer to the Data Availability Statement in the manuscript PDF file). The data should be provided as  part of the manuscript or its supporting information, or deposited to a  public repository. For example, in addition to summary statistics, the data points behind means, medians and variance measures should be available. If there are restrictions on publicly sharing data--e.g. participant privacy or use of data from a third party--those must be  specified.  Reviewer #1: Yes  Reviewer #2: No | This paper is based on secondary analysis of Uganda DHS data that is already available in public domain (<https://dhsprogram.com/data/> ).  Data for this analysis has been attached on the PLOS One depository |
|  | 4. Is the manuscript presented in an intelligible fashion and written in  standard English?  PLOS ONE does not copyedit accepted manuscripts, so the language in  submitted articles must be clear, correct, and unambiguous. Any  typographical or grammatical errors should be corrected at revision, so  please note any specific errors here.  Reviewer #1: Yes  Reviewer #2: Yes | Thank you |
|  | 5. Review Comments to the Author  Please use the space provided to explain your answers to the questions above. You may also include additional comments for the author, including concerns about dual publication, research ethics, or publication ethics. (Please upload your review as an attachment if it exceeds 20,000 characters) |  |
|  | Reviewer #1:  i) The paper is well written and highlights the utilization of health facilities by youth, However, the authors seem to have laid a background on women, not youth specifically. The problem among this  group need to be more pronounced. The methods have been well described. | We have reviewed the background of the study and highlighted the problem among unmarried youth |
|  | ii) Were there limitations since this was secondary data? How was this overcome? | We have included the limitations of the study in the manuscript |
|  | iii) The title could be changed to predictors instead of factors since that is what is entirely referred to in the whole paper. | The title has been changed to read as ‘P**redictors of health facility delivery among unmarried and married youth in Uganda’** |
|  | iv) The results need to have n values. | We have included the n values as the last column in the results tables. |
|  | v) Line 260, correct ANV to ANC. | This has been corrected |
|  | vi) Line 280,  add full stop. | A full stop has been added |
|  | vii) The discussion is well done, it compares the findings well with other studies and also there is the author's attribution of the findings. | Thank you for the complement |
|  | viii) The section on conclusion includes recommendations and it should be titled so. | This has been labeled as conclusion and recommendations |
|  | Reviewer #2: I recommend the authors to use "multilevel logistic regression" analysis since there are community and individual factors rather than using "logistic regression". See the attached files for the detail. | The rationale for using binary logistic regression rather than multilevel regression has been given in number 7 above |
|  | 6. PLOS authors have the option to publish the peer review history of  their article (what does this mean? [3]). If published, this will include your full peer review and any attached files.  If you choose "no", your identity will remain anonymous, but your review may still be made public.  DO YOU WANT YOUR IDENTITY TO BE PUBLIC FOR THIS PEER REVIEW? For  information about this choice, including consent withdrawal, please see our Privacy Policy [4].  Reviewer #1: YES: Joyce Jebet Cheptum  Reviewer #2: YES: Maru Mekie | Thank you for reviewing our manuscript |
