# Supplementary material for: Predictors of health facility childbirth among unmarried and married youth in Uganda
Source: PLoS One. 2022 Apr 7;17(4):e0266657. doi: 10.1371/journal.pone.0266657 (PMC8989320; doi:10.1371/journal.pone.0266657)
Supplement: Attachment — Submitted filename: Review comments - PLOS ONE.doc [file pone.0266657.s003.doc]

**PLOS ONE: Proofs corrections**

**Manuscript No.: PONE-D-20-31194R1**

**Article title: PREDICTORS OF HEALTH FACILITY DELIVERY AMONG UNMARRIED AND MARRIED YOUTH IN UGANDA**

| **Section** | **Comment** |
| --- | --- |
| Title | The title looks good. It has been revised appropriately |
| Abstract | The abstract looks good and clearly highlights the study. |
| Key words | Include predictors. |
| Background | This section is now well done. |
| Materials and methods | The methodology is well written. |
| Results | The results are now well described. |
| Discussion | The discussion is well written with a good comparison with the findings from other studies. |
| Conclusion & Recommendations | The conclusion and recommendations is well written |
| References | This is well done |
| General comments | This is a well written paper with few minor corrections. Check the grammatical errors and correct. Change delivery to birth as it is the new term. |
