# Supplementary material for: Predictors of health facility childbirth among unmarried and married youth in Uganda
Source: PLoS One. 2022 Apr 7;17(4):e0266657. doi: 10.1371/journal.pone.0266657 (PMC8989320; doi:10.1371/journal.pone.0266657)
Supplement: Attachment — Submitted filename: REVIEW PLOS ONE_MM2.docx [file pone.0266657.s004.docx]

REVIEW PLOS ONE

TITLE: FACTORS ASSOCIATED WITH HEALTH FACILITY DELIVERY AMONG UNMARRIED AND MARRIED YOUTH IN UGANDA

ARTICLE ID: PONE-D-20-31194R1

**General comment**

The authors tried to address all the issues raised. Hence, I would like to thank the authors for taking comments to improve the manuscript.

**Issues to be cleared**

**Abstract**

1. It would be good if the authors consider revising the result presentation to make it sound (It is good to compare findings among married and unmarried youths. But the presentation is not attractive and there is redundancy (“Versus” repeatedly mentioned).

Please try to present the result in abstract in reader friendly manner.

**Main document**

**Methods**

2. The authors tried to address the previous comments raised in the method section. However, the are other issues that shall be cleared. What study design have you used? The authors reported nothing regarding the study design used in the manuscript except describing the sampling technique used as well as the type of survey in the DHS data.
3. You have tried to compare the level of facility delivery among married and unmarried youths and the associated factors. Is it a comparative study? If so, is the analysis method appropriate?
4. If it is not comparative study there may not be a need to put the title as “Married and Unmarried youth”. Please clearly address these issues.

**Results**

5. Pages 9-10 in narration of the result as well as pages 10-12 in Table 2; please correct P = 0.000 to “P< 0.001”,
6. You are comparing facility delivery among married and unmarried youth but nothing has been stated about the proportion of married and unmarried youth in each of the variables in table to aside from putting “p” from cross tabulation. Please put the cross-tabulation findings by inserting additional columns in Table 2 “Married youth N(%) “ and Unmarried youth N(%) for each variable”
7. On pages 16, line 329, Table 3: “Multivariate analysis of factors….” Shall be corrected as “Multivariable analysis of factors…since the two terms.
8. Please consider to put the proportion of married and unmarried youth in each of the variables entered in the multivariable model in Table 3 (using cross-tabulation) so that it will be simple for readers to observe consistency as well as to compare variation in the proportion among married and unmarried youths (Example parity one among unmarried & Married, Parity two among married & unmarried …secondary education among married and unmarried etc.) It is a must to have an additional two columns in Table 3 for proportions of unmarried and married youth in each of the variable.
9. On pages 16-17, Table 3, you have put keys for

- RC: Reference Category
- CI: Confidence Interval

However, both are not listed as acronyms in Table 3 . Better to remove

Put 1: as reference category below Table 3.

**Discussion**

10. Page 19, lines 378-380 you explained “Unmarried youth were observed to use health facilities more than the married youth in Uganda between 2006 and 2016. This could be due to the confounding effect of higher parity as most married youth were pregnant for the second time or more.

However, nothing has been stated regarding the proportion of “parity among married and unmarried youth” either in the descriptive part of the result section or another area which makes difficult to confirm whether proportion of parity was higher among unmarried youth compared to the married one.

**Conclusion and recommendation**

Well written and all the comments are addressed.
