# Supplementary material for: Predictors of health facility childbirth among unmarried and married youth in Uganda
Source: PLoS One. 2022 Apr 7;17(4):e0266657. doi: 10.1371/journal.pone.0266657 (PMC8989320; doi:10.1371/journal.pone.0266657)
Supplement: Attachment — Submitted filename: Response to Reviewers.docx [file pone.0266657.s005.docx]

REVIEW PLOS ONE

TITLE: FACTORS ASSOCIATED WITH HEALTH FACILITY DELIVERY AMONG UNMARRIED AND MARRIED YOUTH IN UGANDA

ARTICLE ID: PONE-D-20-31194R1

We appreciate reviewer one for the positive comments. We have tried to improve the manuscript and the grammatical errors.

**General comment**

The authors tried to address all the issues raised. Hence, I would like to thank the authors for taking comments to improve the manuscript.

**Issues to be cleared**

**Abstract**

1. It would be good if the authors consider revising the result presentation to make it sound (It is good to compare findings among married and unmarried youths. But the presentation is not attractive and there is redundancy (“Versus” repeatedly mentioned).

Please try to present the result in abstract in reader friendly manner.

**Response: We have tried to edit the abstract to make it sound and attractive**

**Main document**

**Methods**

2. The authors tried to address the previous comments raised in the method section. However, the are other issues that shall be cleared. What study design have you used? The authors reported nothing regarding the study design used in the manuscript except describing the sampling technique used as well as the type of survey in the DHS data.

**Response: UDHS 2006, 2011 & 2016 are cross-sectional surveys so cross-sectional design was used**

3. You have tried to compare the level of facility delivery among married and unmarried youths and the associated factors. Is it a comparative study? If so, is the analysis method appropriate?

**Yes, this is a comparative study**

**The analysis method is appropriate because it shows the similarities and differences in the use of health facilities at childbirth among unmarried and married youth**

4. If it is not comparative study there may not be a need to put the title as “Married and Unmarried youth”. Please clearly address these issues.

**Response: This is a comparative study**

**Results**

5. Pages 9-10 in narration of the result as well as pages 10-12 in Table 2; please correct P = 0.000 to “P< 0.001”,

**Response: This has been edited on page in line 197-204**

6. You are comparing facility delivery among married and unmarried youth but nothing has been stated about the proportion of married and unmarried youth in each of the variables in table to aside from putting “p” from cross tabulation. Please put the cross-tabulation findings by inserting additional columns in Table 2 “Married youth N(%) “ and Unmarried youth N(%) for each variable”

**Response: The first four columns in the previous table showed the descriptive distribution of unmarried and married youth but may be it was not clear. We have edited it as you suggested; page 10-11**

7. On pages 16, line 329, Table 3: “Multivariate analysis of factors….” Shall be corrected as “Multivariable analysis of factors…since the two terms.

**Response: This has been changed to multivariable**

8. Please consider to put the proportion of married and unmarried youth in each of the variables entered in the multivariable model in Table 3 (using cross-tabulation) so that it will be simple for readers to observe consistency as well as to compare variation in the proportion among married and unmarried youths (Example parity one among unmarried & Married, Parity two among married & unmarried …secondary education among married and unmarried etc.) It is a must to have an additional two columns in Table 3 for proportions of unmarried and married youth in each of the variable.

**Response: I have included the proportions of married and unmarried youth who have had a childbirth from the health facility; page 13-15**

9. On pages 16-17, Table 3, you have put keys for

- RC: Reference Category
- CI: Confidence Interval

However, both are not listed as acronyms in Table 3 . Better to remove

Put 1: as reference category below Table 3.

**Response: RC & CI have been deleted and 1 used as reference category**

**Discussion**

10. Page 19, lines 378-380 you explained “Unmarried youth were observed to use health facilities more than the married youth in Uganda between 2006 and 2016. This could be due to the confounding effect of higher parity as most married youth were pregnant for the second time or more.

However, nothing has been stated regarding the proportion of “parity among married and unmarried youth” either in the descriptive part of the result section or another area which makes difficult to confirm whether proportion of parity was higher among unmarried youth compared to the married one.

**Response: In the descriptive section, we showed the distribution of unmarried and married youth by parity; most unmarried youth (85.6%) were of parity one while most married youth (60.6%) were of parity two, line 178-181**

**Conclusion and recommendation**

Well written and all the comments are addressed.
